# Mind versus machine: can biological models of the mind inspire machine intelligence design?

**DOI:** 10.3389/frai.2026.1894859

**Published:** 2026-09-25

**Authors:** Michael Sharwood Smith

**Affiliations:** School of Management and Languages, Heriot-Watt University, Edinburgh, United Kingdom

**Keywords:** activation, working memory, schemas, cognitive control/executive function, consciousness, GenAI, mind-brain distinction, modular cognition

## Abstract

Models of human cognition that look biologically plausible might in principle be a source of insights that could prove applicable to machine intelligence. The Modular Cognition Framework fits that description in terms of its biological credentials and its potential to identify the challenges confronting GenAI developers. However, it is an account of cognition in the mind and not in the brain. Accordingly, the account includes a clear delineation of the two intimately related concepts, mind and brain, and a definition of their relationship. If either brain or mind can contribute to this debate, a model of the mind could offer a more immediate promising source of inspiration than its complex neural underpinnings. Key features of the theoretical framework include its fundamentally heterarchical architecture having no single locus of control. In addition, general and domain-specific processing principles permit rapid, highly flexible, adaptable interaction between its network of modular systems as they collaborate in the execution of multiple tasks in a constantly changing environment. Eleven types of knowledge are constructed from experience using a similar number of inherited toolkits. Questions are raised as to whether the adaptable and highly creative character of evolved intelligence is reproducible in artificial systems, the best strategies for deriving benefit from studying biological systems and whether neural organisation is really the only place to look for biological insights.

## 1 Introduction

One biological approach to the mind, the *Modular Cognition Framework* (MCF), will be outlined as a potential source of insights for those seeking inspiration for improving machine intelligence (Truscott and Sharwood Smith, 2019; Sharwood Smith, 2026). It comes with a particular definition of the mind/brain relationship. Over a period of several decades, the search for inspiration has generated both positive and critical commentary (Hassabis et al., 2017). More recently there have been claims that an overconfident attribution of superhuman abilities to Gen AI promotes a lack of understanding in users about its limitations (see Crockett and Messeri, 2025; Crockett and Messeri, 2026). Prior to the arrival of GenAI it was safe to state that AI was superior in carrying out specific tasks for which it had been designed but lacked the adaptability and range of skills possessed by humans. With its ability to generate statistically similar outputs on the basis of its training data, GenAI certainly gives the appearance of creativity. At the same time, considering the ease with which people unthinkingly ascribe human-like feelings and attitudes to animals, an overconfident attribution of superior intelligence to AI is a predictable response. In this light, in-depth assessments are required concerning similarities and differences between what humans and machines can accomplish. An account such as one to be presented here can provide a useful and novel type of source for detailed evaluations of the two types of intelligence and the degree to which biology can inform AI research.

The theoretical model selected for discussion, the MCF, makes a clear distinction between the brain and the mind. This clears the way for a separate, detailed and arguably more informative description of how the mind is organised that is entirely separate from a description of its neural underpinnings.

It is important to emphasise the fact that this particular approach to the mind is called a theoretical ‘framework’ rather than a full-bodied theory; it has an open-ended architecture design that permits continual updating and elaboration by researchers in different fields investigating one or other of the eleven designated areas of human cognition. The only condition is that the contributing research is compatible with MCF basic principles as will be outlined in following discussion. These theoretical principles themselves are derived from contemporary trends in cognitive science along with some long established concepts in general psychology and incorporated into a broader picture of processing that includes principles that depend on what type of knowledge representations are being processed. This means that whilst the choice of eleven systems is a special characteristic of the MCF for reasons just mentioned, as well as the particular ways in which it integrates various contemporary research trends, its main principles and concepts still maintain a foothold in a fairly long tradition of theoretical and experimental investigation.

The eleven areas are represented by fixed number of modular systems—‘expert systems’ felt to be the minimum number to explain human cognition. These systems such as the visual system, the affective and conceptual systems, to name but three, form the eleven keystones of the framework architecture. In addition, the framework specifies two types of operating principle: (a) *general* processing principles, well attested in contemporary psychology such as association, activation, competition and storage (memory) and, in addition, (b) eleven *specialised* principles, each unique to one or other of the mind’s eleven systems and reflecting eleven types of biological inheritance. The basic architecture and operating principles of the MCF mind are not ‘designed’ by humans like AI but reflect what might be called ‘evolved intelligence’ (EI), the product of evolution. For this reason the prime task of the MCF is to describe and explain the nature of the brain’s ‘biological software’ rather than the neurological ‘hardware’. This places the psychological explanation of the mind and its neurological counterpart as two parts of one biological explanation whose aim is to characterise the psychological and neurological properties that together define the human species.

The interaction between the (putative) eleven cognitive systems during mental processing allows for the immediate creation and deployment of a limitless number of associations in the form of *schemas*. These associative schemas drive the execution of a huge variety of complex tasks that the mind is faced with, many of them executed at the same time. Processing is assumed to be both incremental and parallel. This explains the human mind’s capacity for rapid, flexible resolution of problems: this is made possible by what could be described as a relatively simple architecture, simple, that is, when compared with the complexity of central nervous system.

The question of exactly *how* an approach such the one to be demonstrated might be applied to improving machine intelligence is a question best answered in detail by those with the appropriate level of expertise but there will be some relevant discussion in the closing sections. The main discussion, however, will concentrate on the MCF, explaining the mind’s internal organisation and operating principles and how the mind achieves what it does by only requiring of the brain sufficient energy per day to power a dim light bulb in order to apply solutions to complex problems, updating old solutions and creating new ones without, for instance, requiring in advance vast datasets to train it and, for most of the time, working online with its various inherited resources to process and interpret experience.

In the first section, the discussion will cover the relationship between the mind and the brain. This will be followed by a brief introduction to the MCF before going into more detail concerning its architecture and operating principles. The third, general discussion section will briefly consider GenAI and human, biological intelligence ending with some concluding observations.

### 1.1 Mind and brain

The MCF, as is appropriate for any attempt to describe and explain the mind, requires a specification of the precise relationship between mind and brain. This is not always provided. Are they one and the same thing? Is ‘brain function’ the same as ‘mind function’? Are they separate as is suggested by Cartesian dualism and criticised by Ryle (1949) or are they closely related but not identical, and if so, how? Not only the social media but also the research literature reveals a disturbing lack of clarity about how these concepts are being treated. Brain and mind seem to be used as substitutes for one another: questions about the mind are often followed by a search for answers from neuroscience. There may well be one dominant view nowadays that is simply so widely taken for granted that it does not merit discussion: in any case, one would like to know when reading relevant publications what the authors are assuming.

The MCF treats the relationship between mind as a brain as relayed but different: as already indicated they are treated as two parts of one biological account defining what it is to be human. The brain side of the story explores the physical side, the anatomical and physiological features that together characterise the human central nervous system. This can be compared to Marr’s third implementation level of analysis (Marr, 1982). The mind side defines the set of properties shared only by humans that together characterise their overt behaviour, It also details their inner states that are open to conscious inspection thanks only to the fact that humans have developed the ability to reflect on and, by using various techniques, to study their own thought processes: this may include forming hypotheses about possible subconscious influences on both their internal states and their outward behaviour. Inevitably some aspects of the brain side of the account, namely the various autonomic ‘life-support’ processes, are relevant but much less so than other subcortical areas and the cortex itself.

The software/hardware analogy is a potentially misleading metaphor chosen here to distinguish the biological accounts of mind and brain. Because this metaphor can easily lead to misconceptions about how the mind is being portrayed, the MCF’s ‘evolved software’ description referred to in the introduction protects it from any incorrect inferences that human minds are scarcely different from human-designed (digital or quantum) computers. ‘Mindware’ and ‘brainware’ are alternatives and perhaps safer terms for the distinction. Freed of misleading implications, the analogy neatly captures the intimate and biological relationship existing between mind and brain and allows us to accept as a fact that an idea or a feeling in the mind is just as real as a population of neurons or a burst of neural activity in the brain.

### 1.2 Relating the twin perspectives

The brain and mind are treated here as two closely related perspectives, both aimed at explaining the essential nature of *homo sapiens*. Their relationship is nevertheless based on two distinctly different types of description and explanation. For example, in tandem with accompanying electrical and chemical activity in the cortex, there is ongoing activity of a different kind in the mind. Not surprisingly given their intimate relationship and notwithstanding the requirement for different definitions, some of the terminology is shared, with ‘networks’, ‘spreading activation’ ‘competition’, and more generally, ‘processing’ being prime examples.

Describing activation in the mind is carried out in terms of its evolved architecture and operating principles. On occasions, despite the temptation to directly link particular patterns of neural activity with mental concepts, such analogies come with cautionary advice not to seek simplistic equivalences without good evidence. The names for the eleven modular systems despite their more elegant description, also relate broadly to the names of equivalent systems distributed across the brain. In sum, the best and simplest way of charactering the mind/brain relationship is as ‘two sides of the same coin’ since each side of a single coin typically needs a separate description and explanation (Sharwood Smith, 2026, pp. 1–5). It would also seem to be a good strategy to develop the two accounts in a manner that they can be fully related as research in both fields advances and take advantage of current of psychological and neurological evidence that is presented in support of modular approaches in general.

### 1.3 Language and mind

The MCF has been developed over the last two and half decades. Initially, the aim was to account for language, the use of which happens to activate very large areas of both mind and brain. In fact, this widespread involvement of language makes it a natural point of entry to studying the mind in general. The first framework-related publication appeared in 2004 and several books and over forty article-length publications have appeared since then (Truscott and Sharwood Smith, 2004).^1^ The aim has always been to integrate as much as possible leading trends in different strands of cognitive science in line with the modular assumptions of the MCF. In so doing, it draws on a range of theoretical and empirical studies carried in different research domains. Since the MCF was originally conceived to account for how language is embedded in the mind as a whole, much of the research thus far has been in this particular area. It is worth pointing out that probably more work than in any other disciplines has gone to researching detailed cognitive structures in the mind as opposed to their equivalent physical manifestations in the brain, especially with regard to grammatical properties, a notable example being the research carried out in various branches of generative linguistics (Chomsky, 1995; Jackendoff, 2002).

## 2 MCF architecture

In this section, the architecture of the MCF will be described with respect to how a modular approach with systems each of which are in most respects autonomous and with specialised representations that never leave their store can nevertheless achieve an impressive degree of *interconnectivity*. It will describe how networks of association are formed and remain with shorter or longer durations running via the fixed network of connecting *pathways* that link the modules. The section will conclude with ways in which *activation* works and an introduction to the ‘preinstalled’ i.e. *inherited toolkits* with which each system constructs and manages its own representations. If one were building a humanoid robot on the basis of the MCF, one of the things that would be necessary would be to attempt designing and installing equivalent toolkits.

### 2.1 Modularity

As indicated in its name, the MCF has a modular architecture. Modularity happens to a dominant feature of contemporary AI. Apart from its practical uses in AI, it shares one very important characteristic with many approaches to human cognition, *namely parallel architecture* allowing work on different types of (sub)tasks to be accomplished simultaneously. In both AI and human cognition circles, there are also opposing points of view and positions in between reflecting for example stricter and more flexible views of modularity. Connectionist and dynamical systems approaches deny any need for modules (Rumelhart and McClelland, 1986; Thelen and Smith, 1994; Kelso, 1995). Within the modularity camp there are different approaches to how many modules and which aspects of cognition should be catered for ranging from fewer in the case of Fodor’s proposals through to massive modularity espoused by Cosmides and Tooby and Carruthers, for example (Cosmides and Tooby, 1994; Carruthers, 2006; van Gelder, 1998). However the MCF reflects a modular approach that is radically different from, for example, that proposed by Anderson et al. (2004). The number of modules that together run the MCF mind are eleven in number and all operate in an identical manner. This is felt to be the smallest plausible number handling eleven distinct types of knowledge (cognitive structure). This does not rule out increasing the number of systems or, less radically, creating submodules, if there is a very good evidence to suggest this and without necessarily embracing massive modularity (Carruthers, 2006): musical, mathematical abilities and face recognition, for example, will always be tempting candidates for some kind of expansion of the network of systems. However, being theoretically parsimonious by sticking to the absolute minimum has the advantage, in the present context, of demonstrating how even this relatively modest number of modular systems and the ways in which they collaborate can achieve the capabilities commonly associated with the human mind: immense flexibility and adaptability in often rapidly changing contexts. The combination of eleven specialised systems, in other words, already yields extraordinary processing power. In addition, extending modularity to non-perceptual systems and thereby removing any question of a central, domain-general cognitive system, is an elaboration of Jackendoff’s general approach to cognition and in particular his architecture of language and his approach to musical cognition (Jackendoff, 1987; Lerdahl and Jackendoff, 1983). In this sense, although it has different way of explaining the ‘workspace’ idea, the MCF comes much closer to global workspace approaches (Baars, 1988; Dehaene and Naccache, 2001).

The eleven systems can be best conceptualised as two concentric circles called ‘rings’. The outer ring has, randomly distributed around it, five sensory perceptual systems interpreting five types of sensation. Each specialised system has a store which contains its interpretations in the form of ‘representations’ of what has been sensed. The five systems, in other words, each build one of a total of five different types of perceptual representation. The physical inputs therefore trigger two types of responses, electrochemical responses in the brain and interpretative processes in the mind. From this initial point, the processing route splits two ways. The MCF is concerned with the second route. The mind’s outer ring systems are, respectively, the *visual, auditory, gustatory, olfactory* and *somatosensory* systems (see Figure 1). These mental systems also reflect the five sensory systems in the brain and, with the exception of the somatosensory system which includes more than just taste, the names of the traditional five senses: sight, hearing, taste. What the rings show are actually the *stores* of each system as will explained below.

**Figure 1:**
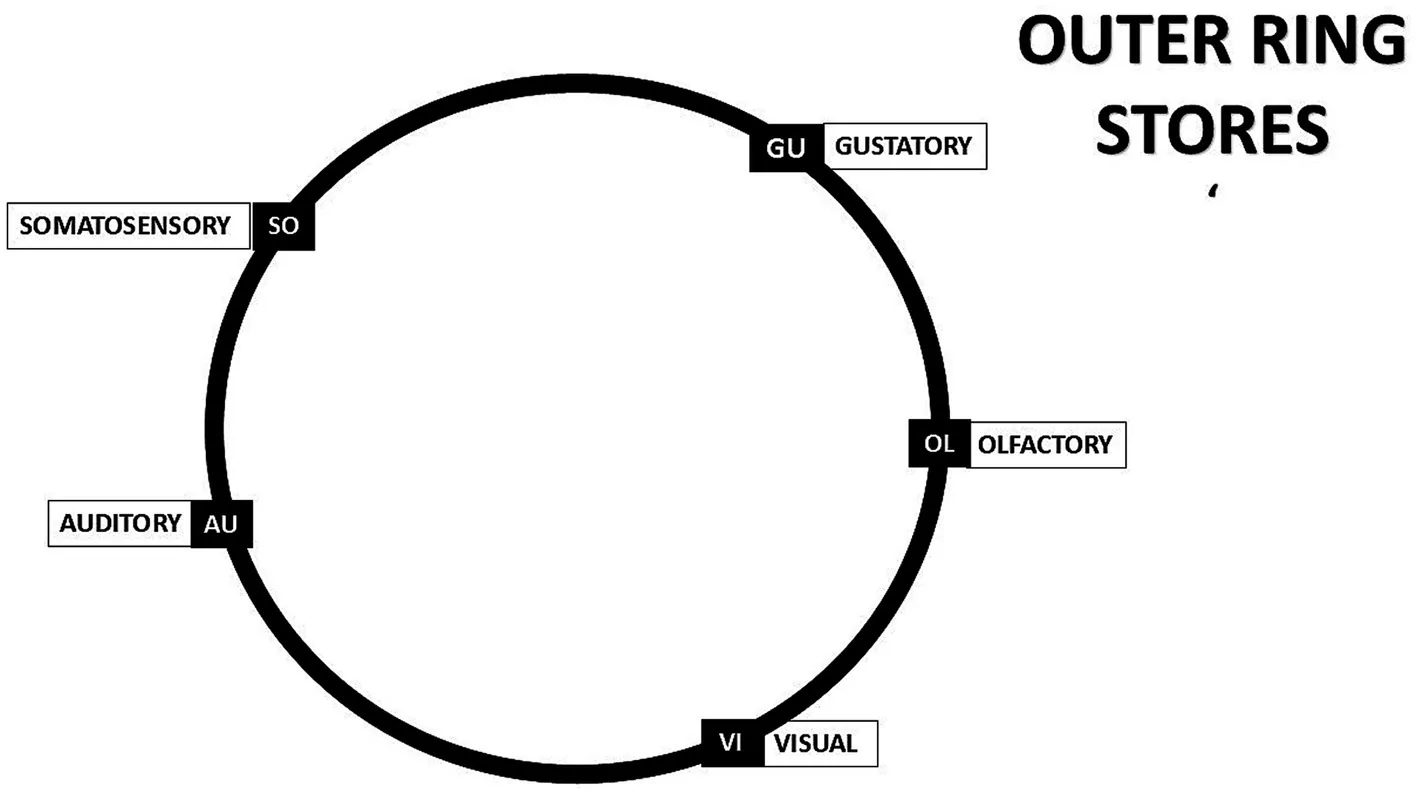
The mind’s five sensory perceptual stores.

The mind’s (as opposed to the brain’s) auditory system for instance builds and handles online auditory representations encoded following its own unique set of principles. These develop over the lifetime a human individual’s internal sound world. This means that when you hear a sound, you are not directly experiencing the raw acoustic signals themselves but indirectly via the internal representation of those signals that the (mental) auditory system has constructed for you. The processing of sensory input originating in the physical environment, in this case the mind’s response to the acoustic input, is very different from the brain’s. Sensory experience in the mind is encoded in five different ways by the systems signalled on the outer ring and, together, the contents of the five stores constitute our internal world, that is to say, our *internally perceived* ‘representation’ of the physical world. Unlike terms like ‘activation’, representation is specifically a mind term so it is not, therefore, legitimate except informally, as a convenient shorthand description, to say that the brain ‘represents’ sensory experience. Representation is the job of the mind: what the brain does is to supply the physical mechanisms to enable that mental representation. The resulting patterns of brain activity, when recorded on a screen for example, are therefore best conceptualised as the physical footprints of mental events. When not talking specifically about brain anatomy and strictly physiological processes in the brain, neuroscientists regularly use such ‘shorthand’ descriptions because they are interested in associating, respectively, neurological and psychological phenomena. This practise remains misleading wherever the current underlying assumptions about how brain and mind relate are left unclarified. To sum up, the outer ring of system represent the closest the mind comes to engaging with the physical environment by receiving as input its physical signals. The signals themselves are processed at the brain level of explanation, However, the (mental) cognitive processing taking place in the outer ring systems represents a lower level that the level of processing than takes place in the inner ring where no direct links with the outside environment exist. As far as the enhancing the interpretation of this environment, the inner systems depend in input that is already internal, namely perceptual representations constructed in the outer ring. This characterises the systems on the inner ring together as a deeper level of processing. Note that, given the modular nature of MCF architecture, ‘input’ from one module in input only in the strictly limited sense that it can trigger association and subsequent coactivation to occur with elements in an adjoining module. Also, the relationship between the two levels, might recall Craik and Lockhart’s classic 1972 paper on levels of processing which in all other respects is not comparable.

The inner ring, in the current visualisation of the brain’s software, contains the stores of the remaining six systems that is to say, respectively, the *conceptual, affective, spatial, syntactic, phonological* and *motor* stores (see Figure 2). These six systems, as just indicated, effectively ‘enrich’ the perceptual information provided by the five perceptual systems. The enrichment is reflected in a web of associations formed between stores on the inner and stores on the outer ring and between the six inner ring stores themselves.

**Figure 2:**
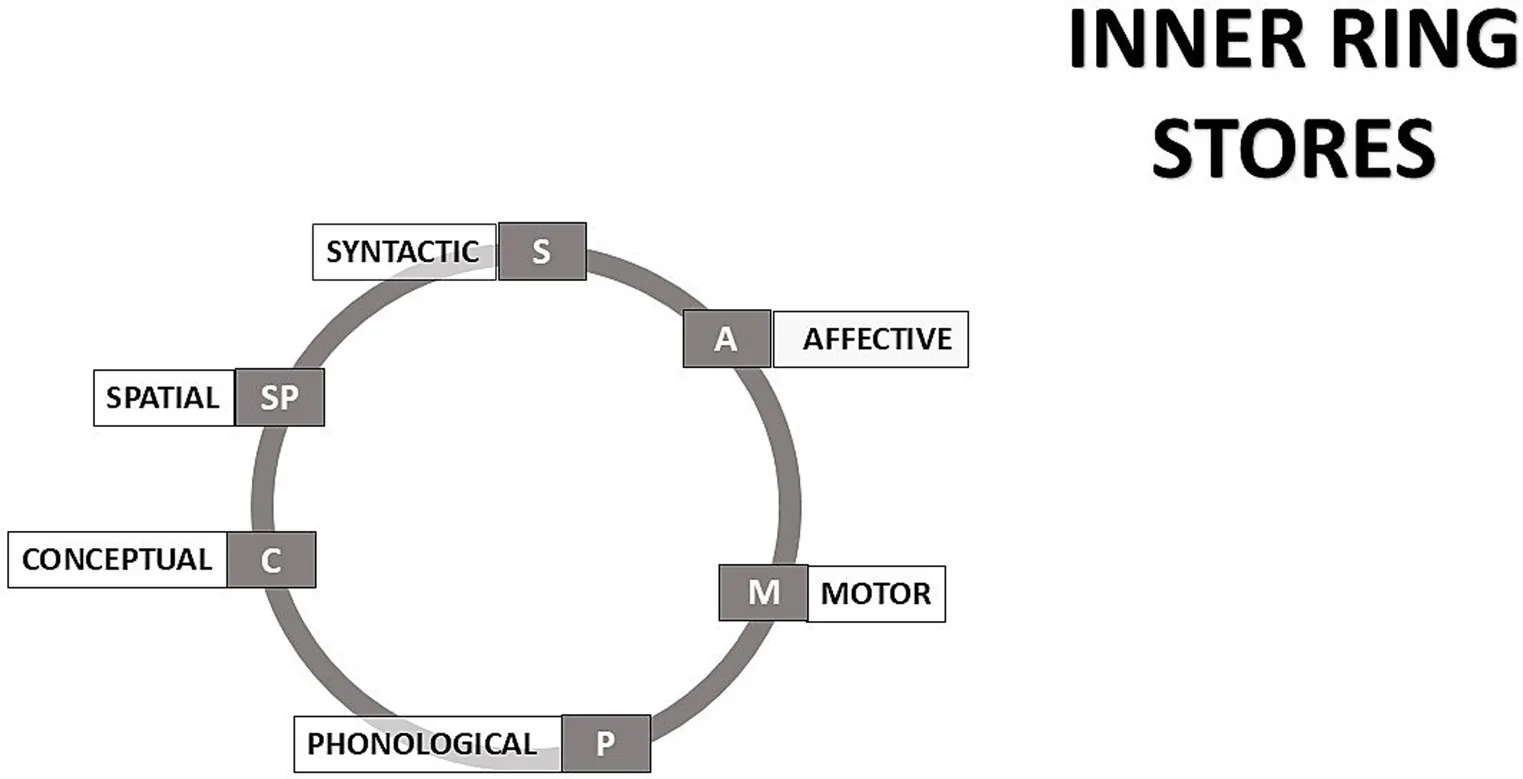
The mind’s inner ring.

As already implied, the eleven systems in the mind have a basic setup of a *processor* and a *store*. The processor incorporates the specialised principles used for constructing a representation in a given system. In the above example they were the auditory principles by which (human) auditory representations are constructed from acoustic experience and maintained in the auditory store. An auditory representation encoded according to these principles is therefore a unique type of structure both with reference to representations in other stores and, in principle, with reference to their equivalents in geckos, grey wolves, gorillas or any other species sensitive to sound. In the present context, an auditory representation underlies a particular type of sensory experience that humans have. To become meaningful, such a representation requires associations that have been formed with conceptual representations in the conceptual store. For example, a particular taste representation in the gustatory store is literally meaningless without any conceptual associations formed via the pathways connecting its store with the conceptual store on the inner ring. Then it not only represents the sensation of tasting but also coactivates associated meaning representations such as such as sweet, familiar, orange etc. We can assume any existing representation is highly likely to have many associations, an observation that naturally leads to a discussion of the mind’s interconnectivity in general.

### 2.2 Interconnectivity

Modular systems are sometimes portrayed as completely encapsulated and severely limited in how they can interact with each other (see for example Fodor, 1983). In the case of the MCF, the stores of is eleven systems are richly interconnected in a manner that allows collaboration with other modules whilst retaining their status as modules by restricting direct access to their contexts.

#### 2.2.1 Association and schemas

There is no obstacle in this modular approach to saying the mind is a massively interconnected network. The question is the *nature* of its interconnectivity. There is no possibility of free-for-all interaction as occurs between nodes in a classical connectionist network. Because they share the same code, representations in one store may be freely associated with one another but, for the same reason, are incompatible with any other type of representation. How then can representations in different stores interact? Collaboration between modules consists precisely in the formation of associations that preserve the modular character of each system. Associations, when active, lead immediately to co-activation: the activation of a representation in one store will *coactivate* any representations elsewhere with which an association has been formed. This process results in webs of associations called ‘schemas’. Participating representations still remain activated *in situ*, so each in its own store in accordance with its modular character. This will have implications for the explanation of working memory (to be discussed in section 2.2).

Effectively, processing in the mind will always involve multiple *schemas* with associations involving many representations being coactivated in their own stores. The general proliferation of schemas as a result of continuing diverse experience means that activation of one representation will typically spread out instantly to many other representations across the whole network. Such large schemas that range across multiple stores are best thought of as *coalitions* rather than combinations of individual representations. Schemas can be stored, activated and modified during online processing. They can also become fragmented with lack of use.

The formation of schemas is made possible via the permanent network of connections that link up the eleven modular systems: as already indicated, these are called ‘interfaces’ or ‘pathways’. Direct associations such as sound-meaning associations can be formed between directly connecting stores but associations can also be indirect in any schema involving more than two stores as can be traced in any of the figures below.

#### 2.2.2 Processing hubs

Indirect associations between representations in different stores arise because not every store is directly connected with every other store. Two important systems stand out in the creation and activation of schemas. The *conceptual* (semantic) store is one which functions as a hub standing at the crossroads of many other systems with many chains of association running in different directions across the two rings do so via an intervening conceptual representation. This gives the store sitting at the crossroads of all these associations the character of appearance of hub with the associations like the ‘spokes’ of a wheel, radiating out to other stores. Meanings are then conceptual representations that stand at the centre of a network whereby conceptual representations are associated with representations located in almost any of the other stores. Not surprisingly, then, the processing of meaning on a given occasion will typically activate schemas that involve most or all of the eleven systems and this spread will also be reflected, albeit in different ways, in accompanying patterns of neural activation. The hub-and-spoke metaphor was in fact borrowed from a broadly comparable approach to conceptual cognition in the brain (Lambon Ralph et al., 2017; Rogers and Lambon Ralph, 2022).

Figure 3 shows the complete set of stores on both rings together with only those specific pathways (interfaces), displayed as double lines, that radiate from the conceptual (c) store. Other pathways are excluded in the figure for convenience just to keep the display simple. Note also that the rings hosting the various stores have no connecting function.

**Figure 3:**
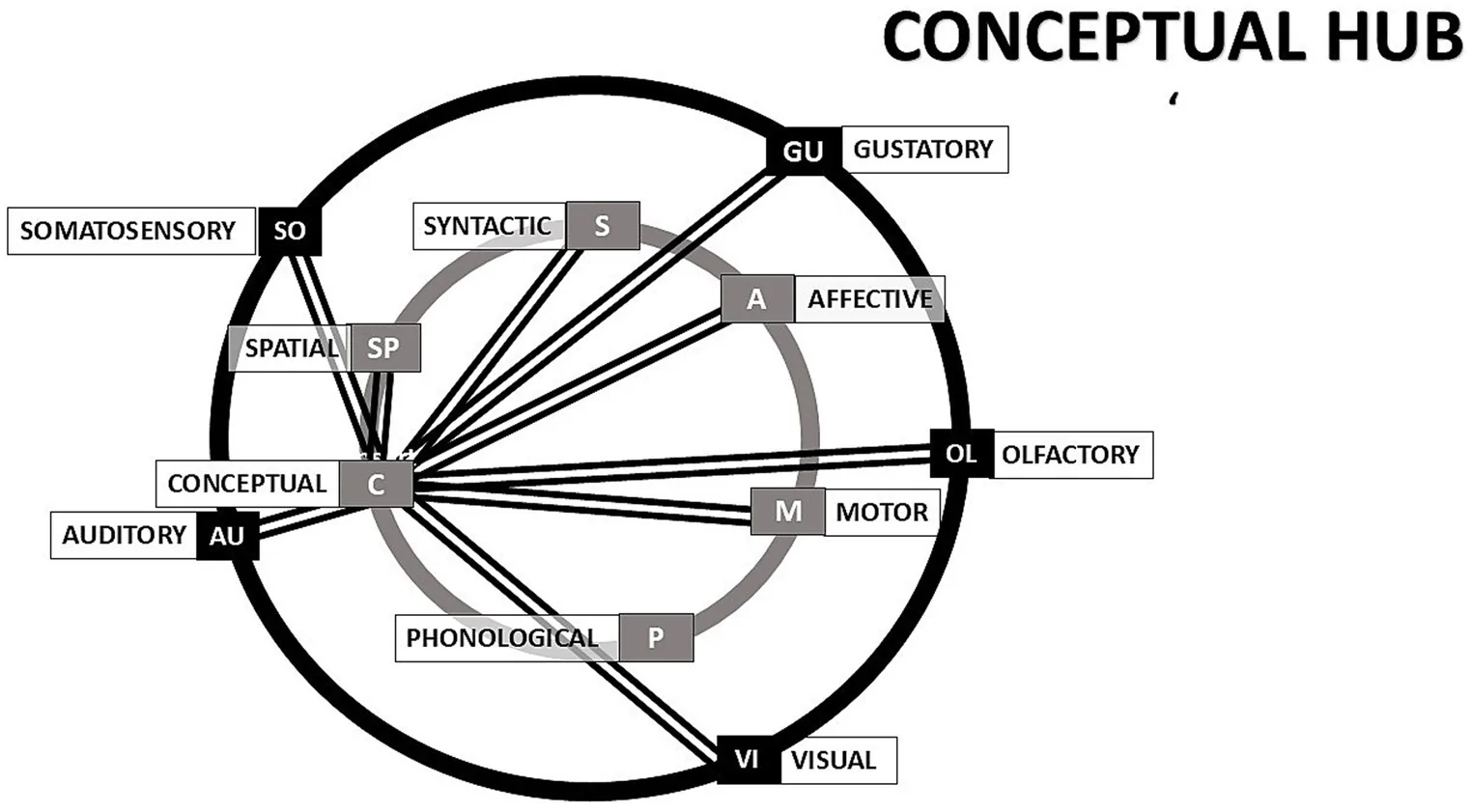
The conceptual ‘hub and spokes’.

As can be seen in the figure, associations in a schema can be direct or indirect. A *syntactic* (s) representation, like N(oun) on the inner ring cannot associate directly with the auditory representation (au) of the sound ‘jump’, say, but it does have a connection with a *conceptual* representation and so can connect to the sound of a word indirectly in a three-item schema as follows: N ↔ jump (meaning representation) ↔ ‘jump’ (sound representation).

#### 2.2.3 Permanent and temporary networks

The system of interface pathways being part of the fixed architecture of the MCF can be contrasted with the essentially *temporary* associations that are formed using these pathways. Figure 4 shows just such a set, i.e., schema of associations (shown with dotted lines) that could remain intact anywhere between a lifetime and a matter of seconds. Like almost all representations, the representations displayed in this figure as single items are actually complex ones, that is to say they are composed of many features. We could imagine a typical representation as being ‘molecular’, a cluster of a number of atomic features, each feature itself being a representation in its own right. Note that this represents a level of granularity far greater than that displayed, for example, by the tokenisation of text in large language models (Su et al., 2026). Also note that, in reality, the schema in Figure 4 would typically have many more associations involving more stores than just the four in this simple illustration (a, c, sp. and vi).

**Figure 4:**
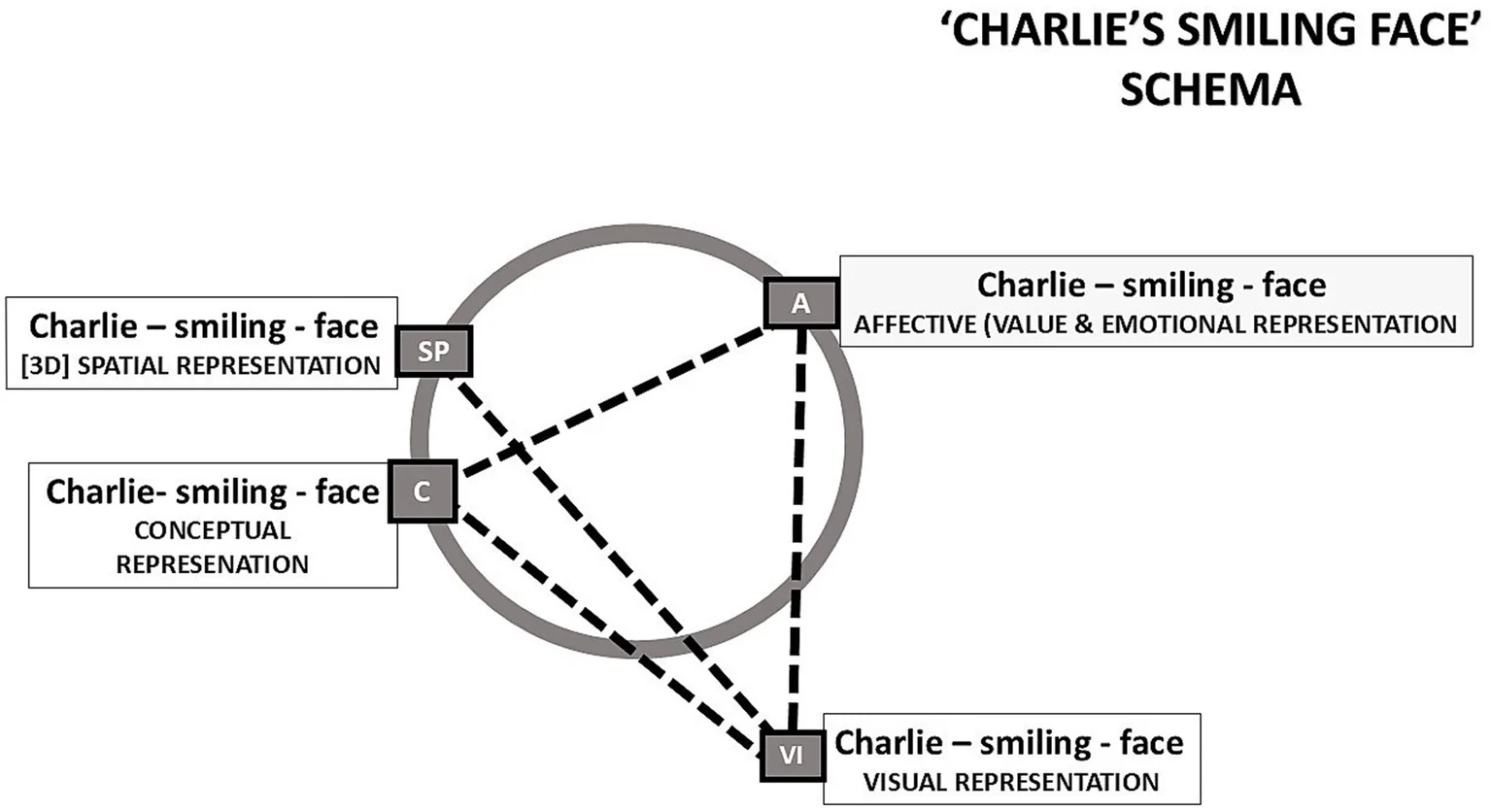
A language schema.

The *affective* (a) *store,* which contains value and emotion representations, also has a hub-like character. It plays a vital role in processing chiefly because of its values: these influence the activation levels of representations and schemas online along with shifts in the current context. In the Figure 4 schema, there are representations in the affective store (a)—a positive value representation, if not on its own then as a component of the complex representation of a basic emotion like ‘happy’. These affective representations are associated with any or all of the meaning representations of ‘Charlie’s smiling face’ located in the conceptual store (c). The affective representations are also associated directly with *visual* representations of Charlie’s smiling face in the visual (vi) store as well. Furthermore, for Charlie’s smiling face to be represented in the current context as a full 3D visual representation, this visual representation needs its spatial properties involved in the schema. Accordingly, it is also associated directly with an appropriate *spatial* representation in the spatial (sp) store (Sharwood Smith, 2026, pp.100–104). Note that only one type of representation in this schema is located in the outer ring, namely in the visual store: the other four representations are located on the inner ring. Such patterns of association would be reflected in corresponding patterns of much greater complexity as neural connections distributed over various regions of the brain.

Figure 5 (below) displays a slightly more complex schema than the previous one. It depicts an existing schema of associations that would be activated in response to the simple two-word, spoken or written sentence: ‘Robins fly’.

**Figure 5:**
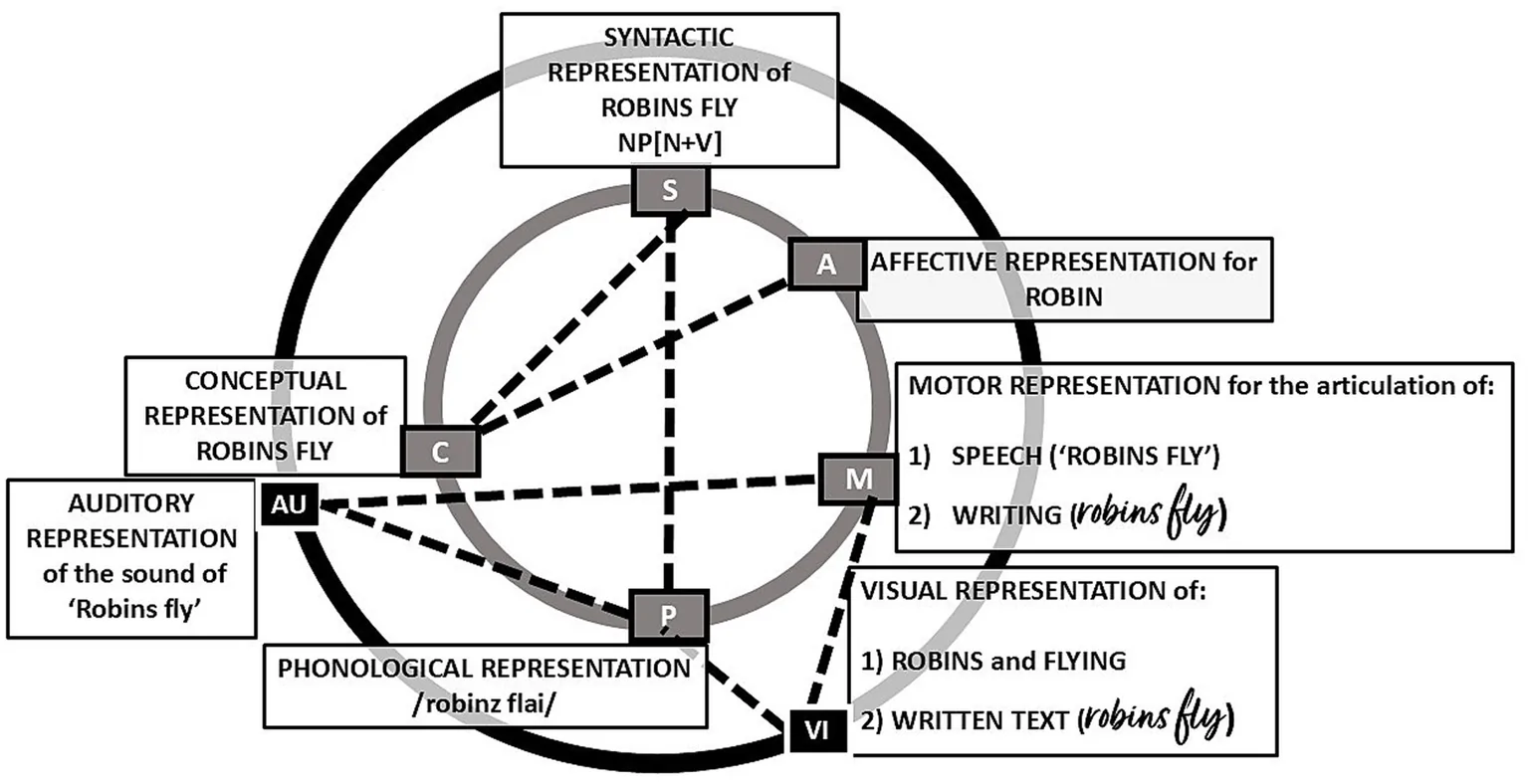
A ‘Robins fly’ schema.

Note that communication between humans can be carried out in various ways and does not have to directly involve language (Sharwood Smith, 2024). The direct involvement in this Figure 5 schema of *linguistic* (syntactic and phonological) structures qualifies the schema as ‘language-related’. However, as is clear from the figure, these two dedicated linguistic stores are only two of a grand total of *seven* stores involved in this language-related schema. Five of them have nothing directly to do with ‘linguistic’ structure as defined by the MCF. For example, two of the stores (vi and au) are on the outer ring and serve only to represent raw signals sensed in the environment irrespective of any relevance they might or might not have for language. The labels in square boxes next to each store in Figure 5 contain informal descriptions and so are devoid of fine technical detail. Nevertheless, this schema should already convey some sense of the (instantaneous) complex response that a simple two-word sentence, ‘Robins fly’, might trigger in the mind and how a modular system can handle this response efficiently.

The concept, ‘language’ is considerably broader that what could be called the basic grammatical component of language which in the MCF is actually a joint contribution of two above-mentioned linguistic modules, one dealing with morphosyntactic structure (s) and the other with phonological structure (p). This separation of linguistic structure into two autonomous modules follows Jackendoff’s architecture of the language faculty (Jackendoff, 2002). These two modules contain primitives that together have been referred as ‘universal grammar’ in the generative literature (cf. Chomsky, 1995) although they do not constitute a grammar in the customary sense but rather contain the two inherited toolkits, from the total of eleven, and which enable the building of complex grammars from experience and for whatever language or languages to which the individual, especially a very young child, happens to be exposed.

Language, now in its widest sense, involves, at one point or other, the activation of representations in virtually every other store in the mind. Figure 5 also shows how the visual (vi) store represents not only visual experiences associated with robins but also the visual experience of a series of marks on paper or screen that the individual will recognise as ‘text’. In the same way, the auditory (au) store only contains the sound representations associated with ‘Robins fly’ but it is the phonological (p) store that supplies its speech structure. Finally, the motor (m) store provides the articulatory ‘programmes’ required to produce, in the form of speech, not only the sound of ‘Robins fly’ but any movements needed to create its manifestation as written text. All these non-linguistic but still language-associated representations are typically co-activated in the schema ultra-rapidly and in parallel.

The conceptual system which handles meanings irrespective of whether a given meaning is not easily expressed, i.e., has no direct match with a word or phrase in any language, is also a *non*-linguistic system. It does, however, play a major role in the semantics and pragmatics of a language. This explains why, as Leivada et al. (2026) point out, that large language modules with massive text repositories, perform considerably worse that humans at processing the distortions (‘noise’) in text characterising ‘leetspeak’ (as used in gaming on the web, for example) like: ‘1 t’5 n1c3 t0 s33 y0u’ (‘it’s nice to see you’). The mind needs and possesses more than just morphosyntax and phonology to interpret language utterances.

### 2.3 Activation and working memory

Thus far, a standard category in processing literature, *memory* has not been mentioned specifically. The MCF adopts an activation approach to working memory (cf. Cowan, 1999; Postle, 2006). This means that the contents of WM consists at any given moment of those representations that happen to be currently in a state of activation. Given its modular architecture, this means that working memory is also modularised. In other words, all the eleven stores contain their own differently encoded representations and, whenever these are activated to become WM, they are activated in their separate stores. The only other type of activation relates to the *associations* themselves that link currently activated representations across stores allowing activation to spread so that whole schemas become active. Accordingly, if we speak of representations ‘in working memory (WM)’ we are effectively talking about the activation of schemas involving more than one, often multiple simultaneous working memories, e.g., *auditory WM, conceptual WM, motor WM*. As far as the concept of ‘long term’ memory (LTM) is concerned, this is rendered unnecessary referring by default to whatever representations, in a resting state, are currently in the possession of an individual, that is to say, accumulated in one or other of their eleven stores each with their various associations. Exactly how long individual representations and schemas stay intact will vary from anything ranging between extremely short to extremely long periods of time depending on their activation history.

### 2.4 Activation a matter of degree

One important point to make about activation is that it is always *a matter of degree*. What determines the degree of activation is firstly the frequency and regularity with which a given representation has been activated. If the activation history of a representation is marked by a decline in its frequency of use, its default level of activation also declines and it becomes less competitive. This effectively pits two processes against each other. The effect of being activated is always that its resting level is raised even fractionally. The more frequent the activation the steadier is the rise in its resting level and therefore in its potential to compete. The decline that always takes place during periods of *non*-activation where these inactive periods may get longer and longer and, if it does, will eventually outweigh any benefit it got from repeated activation in the past.

Processing activity is marked by *competition*, one of MCF’s *general* processing principles and standard in most, if not all accounts of processing: more candidates for the execution of a particular task are activated than will ultimately be deployed to execute it. Efficient problem-solving is achieved by readying a number of potential candidates before one gets to dominate and this activation process is automatic and not contrived by anyone or anything apart from evolution. If a round object is perceived, many representations associated with round objects will initially be activated. This multiple activation can also be thought of as reflecting the rich interconnectivity in the mind: so many representations are associated with other representations and accordingly get coactivated. The longer a candidate remains currently activated, the more it benefits when finally falling back to its new, improved resting level. A losing candidate which has been activated for shorter period and has not participated in the ultimate resolution of the task is assumed to benefit less, returning to a raised resting level that reflects a more modest improvement.

The difference between an activated and a resting state is, in practise, blurred since there is probably a great deal of very minor background activity going on all the time. In principle, though, the difference is clear. Whenever an inactive, i.e., ‘resting’ representation gets activated it rises from its current resting level. The notion of degrees of resting level is the MCF’s conceptualisation of a representation’s current competitiveness, that is, the current potential of a given representation to rise to a given activation level when activated. Lack of use leads to a decline in resting levels and a related decline in that potential up to a point where it seems to become virtually ‘inaccessible’. This is a ‘use it or lose it’ principle. There are other forces at work related to affective value and contextual shifts and which can influence the default competitiveness of a given representation when activated. The present account of activation, although here expressed in the terms of the MCF architecture, is taken to generally reflect a broad base of opinion in contemporary psychological literature.

### 2.5 Biological starter packages

#### 2.5.1 Knowledge in general

First, it is important to establish what ‘knowledge’ is in the MCF perspective which, although it is literally ‘connectionist’ in that sees the mind in terms of networks is based on the mind as consisting of in networks, is also based on the idea of a symbolic representations. These representations represent a higher level of processing than their neural instantiations in the brain.

What, then, is it that we, or more precisely put, our *minds* actually ‘represent’? Traditional notions of mind seem to focus exclusively on what is accessible to awareness, i.e., thoughts. This is the ‘cogito’ in Descartes’ *cogito ergo sum.* It may also include feelings and sensations. Either way, what we are actually at any given moment aware of covers only a small percentage of our mental activity. Maintaining what are purely *consciousness*-based views of the mind is understandable since it is via conscious processing that humans have been able to create our built environment, has guided are thinking and creativity over the millennia and all that has ensued from that, consequences both good or bad. It includes all the knowledge, factual and ideological, that individuals have stored in physical libraries and in cyberspace and which is passed on from generation to generation. Nevertheless, in most contemporary approaches to the mind like the one adopted here, knowledge consists of representations, i.e., cognitive structures a large proportion of which are regularly, sometimes always activated *below* the level of awareness. If we are reasonably assuming the existence of the subconscious, it could hardly be otherwise. Accepting its existence does carry with it the temptation to imagine an independent subconscious mind, some kind of homunculus at work influencing, sometimes undermining consciously formed goals and opinions. The homunculus idea of a mind-within-a-mind idea can be dismissed as it essentially explains nothing and, in addition, even the conscious mind cannot be granted the status of an overall supervisor as much as would like to be (Ryle, 1949; Dennett, 1991).

Summing up so far, knowledge in the MCF consists of at least eleven types of stored cognitive representation which can be divided into those that are *acquired* as a result of life experience and those that are *given i*n advance as part of our biological inheritance. Some of that knowledge can participate in conscious thought. Quite a lot can be active below the threshold of awareness. Part of that subconscious knowledge remains hidden from any kind of direct access whilst still influencing our behaviour: we also have no access to underlying mental processing operations, only their outcomes. It is safe to say, however, that any biological account of the human mind ignores subconscious processing at its peril.

#### 2.5.2 Given knowledge

Since the development of biological knowledge is not completely data driven, the question now is about what knowledge is available to the mind *prior to anything gained from experience*. So far, the discussion has focused on the basic MCF architecture and its general and specific principles for gaining and activating acquired knowledge. The question of ‘preexisting knowledge’, that is what would be, in terms of the brain, part of our DNA, must be specified in any model of the mind that claims to be ‘biological’. Put another way if there is a human genome, there must also be an equivalent human ‘psychome’ which expresses the array of mental properties that humans and only humans, even counting their nearest primate relatives with whom we have so much in common. Given knowledge is, therefore, part of the ‘pre-installed’ software, the pre-installation of course being a metaphor for what is actually the product of evolutionary processes.

#### 2.5.3 Primitives

In the MCF, since all systems are modular, none of them having fixed precedence over the others, each system has available a finite set of features, that is so say, *primitive representations* (‘primitives’) which are used to develop those representations that must be created from an individual’s experience over the lifetime. The (mind’s) auditory system, for example has a set of auditory primitives that form the basis of building any auditory representation for acoustic input. These primitives in various combinations serve to identify its representations as belonging solely to the mind’s auditory systems and no other. This pattern is repeated for all remaining ten systems. The precise characterisation of such primitives is a task for researchers working in the subdomain of cognitive science relevant to one or other of the eleven systems. Explanations and accompanying terminology in the neuroscientific literature of how the primary and secondary visual cortex (V1 and V2) respond to particular visual cues can provide possible material for enumerating visual primitives in the mind. By contrast, compared to any equivalent research on the human cortex, the specification of possible syntactic features that could be classed as syntactic primitives in the mind has already had a long history, particularly in generative linguistics (Chomsky, 1995; Chomsky and Halle, 1968).

#### 2.5.4 Inherited schemas

Thus far, the existence of primitives, single basic elements in each of the eleven systems. Has been proposed. However, as part of the eleven ‘biological starter packages’ there must also have been some preexisting *schemas* whereby primitives in one system have already acquired associations with primitives in another. Taste offers an obvious example of such mini-schemas. The physical response to certain bitter and sweet tastes in the infant cannot have been learned from experience. It is present at birth and, one presumes, is there as an evolved strategy of self-protection against the ingestion of toxic substances. Another response might promote the ingestion of beneficial nutritive substances such as breast milk. In either case, there must be a preexisting link between for example value primitives in the affective store and gustatory primitives so that the bitter or sweet taste is already marked as, respectively, harmful or rewarding, negative or positive. In AI, this inherited knowledge might provide examples of what would have to be programmed into biologically-inspired AI software.

#### 2.5.5 Individual variation

One final thing needs to be added to the explanation of biological toolkits, relating to inherited traits that do not identify the individual as human but rather *differentiate* one human individual from others. These differentiating characteristics can be attributed to the effect of variation in the initial *resting levels* of certain primitives including the initial values associated with them. Recall that a low resting level, for example, means that the representation is handicapped. When activated and, all other things being equal, it cannot rise to a level high enough to compete successfully with other coactivated candidates. These different initial ‘settings’ will be manifested as different levels of behavioural response to given experiences varying from minimal to very pronounced. Relatively stronger or weaker responses to certain *sensory* experiences would be one manifestation of such settings. Individuals will respond differently to certain high-pitched sounds or the feel of certain textures. Another examples of response variation would be reflected in given traits associated with different personality types. To sum up, the inherited toolkits that processors in the eleven systems rely on must include associative schemas that link primitives in different systems and in that way account for individual variation in the strength of responses present prior to any modification driven by life experience.

## 3 Internal context

A major feature of the MCF is the sharp distinction it makes between whatever is happening in an individual’s external (physical) environment and whatever is currently active *within* that individual’s mind. ‘Internal context’ is about what is influencing processing on a particular occasion. This conceptualisation of context is not only relevant for understanding the reality that our minds actually work with but especially because of its role in the explanation of cognitive control.

There are two sources that contribute to current internal context. Firstly, despite the fact that behaviour appears to be influenced directly by states and events in the physical environment it is actually an indirect and very selective response. In ongoing situations, then, it is the individual’s current internal *representation* of external events that counts as part of the ongoing internal context. Secondly and even more importantly, there is activity in the mind that originates not in the outer world but comes from *purely internal* sources. The source of this activity comes from the systems on the inner ring, for example, emotions and values and these provide internal input from within to the outer systems influencing how they represent the external circumstances. You could say, for example that of all acoustic features that you are processing, you still hear those that your mind values and ‘prefers you to see’ This is internally derived context influencing your current perception of external events. In sum, when considering any type of processing activity, what factors might influence its outcome, the answers will always come from the current internal context.

### 3.1 Hierarchy and heterarchy

Earlier it was stated that amongst the eleven interconnected systems of the human mind, not one of them had a fixed precedence over all the others. This makes the organisation of the mind as network of autonomous systems essentially ‘heterarchical’. This also means that there is no single, fixed supervisory system that manages the mind’s activities. It does not exclude hierarchical processing but it marks its fundamentally heterarchical character and implies that the locus of control will shift according to circumstances. Depending on how a current situation is represented internally, a given context will promote particular associations over others. Internal states of mind that are not specifically associated with any particular situation will also influence the outcome in particular ways: a stressed or distracted person will perform differently. One constant factor will be the stabilising influence of the mind’s drive to maintain as state of coherence and stability. In this way, the MCF thus exhibits one key feature of intelligence, namely the ability to adapt rapidly and appropriately to shifts in the context, shift which themselves can be rapid, and it does so thanks to its modular, heterarchical architecture.

## 4 The human conceptual system

Discussion will now focus on of two important systems both functioning as a hub with multiple connections giving it direct access to the stores of most of the of other stores. The first of these two systems is the one that handles *meanings* namely the conceptual system that is located on the MCF’s inner ring.

### 4.1 Knowledge, awareness and value

Since much of what is generally regarded as ‘knowledge’ (ignoring for the moment its more inclusive MCF definition) is actually centred on meanings, i.e., conceptual structures that are created and stored in the conceptual system along with the radiating associations that coactivate representations in other stores. Meaning representations—‘conceptual knowledge’—can appear in the ‘mind’s eye’, an apt expression given the fact that vision is perhaps our strongest sense. The outer ring systems together create a steady background state of awareness in the waking state, important for an individual’s survival but they also permits meanings to be sensed as though they were themselves perceptual representations stored in the outer ring. Even if a comprehensive explanation of the phenomenon remains elusive, higher consciousness, the ability to reflect and analyse, is also provided with an explanation in processing terms by employing activation theory as interpreted within the framework. One prime condition for becoming aware of meanings is, then, that the various representations in the schema that has conceptual representations in its hub must be activated up to sufficiently intense levels. Only then can thinking be carried out in its characteristically slow, linear, and at least in human terms, energy-intensive fashion. The same representations can still be regularly activated and participate in schemas *below* that threshold, rapidly in a non-linear manner. This activation-based account of conscious processing does not of itself explain everything about consciousness but it provides a hypothesis about the mental mechanisms involved. As far as corresponding neural mechanisms are concerned, support from neuroscientific research comes from Dehaene and others supporting his view of global broadcasting whereby, in a sudden burst of neural activity—information is shared across many brain networks—namely, the global neuronal workspace account (Mashour et al., 2020; Dehaene, 2015). Humans are clearly not the only species on this planet that have the ability to become aware of their surroundings. However, to what extent they are able to analyse their situation, reflect on the past and plan ahead does not approach what humans can do. These complex thought processes must owe much to the interaction between a rich accumulated repertoire of concepts in the conceptual store considerably enhanced by the use of language enabling the construction of ever more complex meanings (Sharwood Smith, 2024, p. 111). The degree of activation required from the basic, sensory awareness of our surroundings and by hypothesis shared by other species is still not sufficient for the analytic, reflective awareness and deliberate decision-making that only humans appear to possess.

### 4.2 The modular nature of conceptual knowledge

The conceptual system with its multiple associations with representations in other stores is perhaps the closest the MCF gets to the kind of all-purpose domain-general cognitive system envisaged by those who want to restrict modularity to the perceptual systems, i.e., to what would be the five systems on the MCF outer ring. It also approximates in one sense at least to the declarative memory module in Anderson’s ACT_R modular theory (Anderson et al., 2004). And, at first glance too, the conceptual system comes close to the traditional and narrower notion of the mind based on whatever we can be consciously aware of, judge to be true or false and make deliberate decisions to solve a vast variety of complex problems consciously, in short, as the basis for *thinking*, that is, including both rational and non-rational thought. However, it differs from all of other approaches in important ways, the most important of which is that it is, along with the other ten systems, a modular system in its own right possessing its own dedicated processor governing the construction and manipulation of its own specialised representations and its own store. In particular it is not responsible for handling grammatical structure because has its own way of organising meaning according to its conceptual principles. This marks a key difference between, on the one hand, the MCF approach to language which follows a long established generative linguistic line of thinking and, on the other hand, the other main cognitive approach to language which denies the existence of a special human capacity for language, arguing that grammatical structure can be acquired and used in the same way as any other kind of knowledge following all purpose ‘domain-general’ principles (Christiansen and Chater, 2015). The MCF also has its own domain-general principles that apply across the board but each system has its own (putatively eleven) specialised principles described above along with eleven inherited ‘toolkits’ that help build and manage its eleven types of representation.

### 4.3 The conceptual system and cognitive control

Cognitive control, sometimes referred to as executive function, as investigated in task-switching tests, is a broadly defined concept that must be included in any explanation of the mind and especially in a view of the mind claimed to be predominantly heterarchical with no fixed executive system in charge. Cognitive control should not be thought of as restricted to deliberate (conscious) decision-making. This is because subconscious influences can produce behaviour that runs counter to deliberately conceived goals such as eating less meat, taking more exercise, or liking a person strongly disapproved of, or intuitive responses that lack any rational basis, a phenomenon highlighted in Kahneman’s well-known distinction between fast and slow thinking (Kahneman, 2011).

In the MCF, a major determinant of internal states and overt behaviour is context, that is to say internal context as described above. Walking into a familiar type of situation, a shopping mall, a school, a bank, a formal reception, a job interview, an encounter with a close friend, etc., will cause a cluster of associated schemas that are well-established and with high activation potential to be activated. No conscious decisions are required for this to occur. For this reason a great deal of human behaviour is predictable. However, when something unexpected happens, current behaviour changes. A bomb explodes, everybody freezes for a second (see LeDoux, 2002, p.6). This behaviour is instinctive. The ‘goals’ underlying such instinctive responses are inherited and often termed ‘drives’ rather than goals. They are not goal structures assembled in the conceptual system but, in MCF terms at least, they are part of the affective toolkit of primitives and schemas also implicating, on the brain side, the autonomic system. What follows, then, is a sudden downgrading in value and therefore in activation levels, of schemas that were associated with the previous situation as the interpretation of the new situation (danger) activates schemas with resting levels that are set high enough to dominate any competition.

Value representations in the affective system are its most important primitives: every basic emotion, joy, surprise, disgust, etc., has either a positive or negative value as one of its components. Representations elsewhere when associated with either positive or negative values have their activation levels raised as a result. Spreading activation will cause a representation with a high activation level to raise the activation levels of the other representations in its schema making the schema as a whole more competitive. As in the bomb explosion example above, shifts in the internal context can cause shifts in activation levels: what were, until the second before the explosion, the currently dominating schemas can suddenly be lose out against other schemas associated with the new internal context. Another less dramatic example would be a change in behaviour whilst chatting informally with a friend when joined unexpectedly by someone with whom you have only very formal relations, for example a boss.

## 5 Discussion

The basic questions that have been so far left unanswered are the following: in what ways does the MCF characterise human intelligence that can be usefully compared with GenAI and what conclusions arise from such a comparison that will cast light on the alleged superiority of AI in solving all kinds of complex problems.

### 5.1 Mind, brain and GenAI compared

The desire to improve AI and build on its current successes has stimulated interest in the neural organisation and processing in the brain but not without controversy. Fakhar and Astle (henceforth F&A), for example, adopt a critical stance on the usefulness of the human brain as a source of inspiration. They point out that using human brain architecture as a basis for AI as well as a means of explaining psychiatric and neurological conditions is the wrong way to go (Fakhar and Astle, 2026) They criticise those AI researchers seeking inspiration from the brain for their assumption that the brain’s complex wiring is superior to anything that AI has achieved so far and that therefore designing brain-like AI architectures might not be the best way forward. F&A state that ‘duplicating the brain’s connectivity risks copying its irrelevant compromises’. Mention is made of trade-offs that make brain connectivity suboptimal such as trade-offs between functional efficiency and energy costs, something which might still in itself be regarded as an optimal adaptation for a biological system given the changing circumstances under which the brain has to operate. On the other hand, Chen et al. (2022), for example, recommend persisting with brain-inspired models, proposing their own way of approaching the challenge.

The criticisms made by F&A with respect to neuro-AI thinking seem well founded especially with respect to those who take for granted the value and applicability of brain architecture for improving AI. From the perspective of the MCF comparing brain architecture with AI just seems based on a category error. Given the MCF distinction between brain level and the mind level of explanation, it might be more logical to compare the brain’s neural ‘hardware’ with physical machinery required to run AI such as GPUs. This does not seem the right way to go, however. The frequent confusion of brain and mind or the blurring of this distinction referred to at the start of this discussion seems to be reproduced in this ongoing debate. Ren and Xia (2024), for example, do make a distinction between brain-inspired AI (BIAI) and AI inspired by human behaviour which is suggests something like an acknowledgement of the MCF-based brain-mind distinction. Significantly, they still elect to devote their review to the former source of inspiration, the brain. Whether that choice is motivated by practical concerns due to a perceived scarcity of suitable models of the mind, biologically inspired or not, is not clear.

From an MCF point of view, what is really at issue is which set of biological phenomena should provide the best source of insights for improving (Gen)AI. The logic of the two-level distinction dictates that AI programmes searching for inspiration from biology, have a choice consisting of two options plus a third option: a combination of both, that is, as between accounts directed towards the strictly neural organisation of the human brain and quite different, systematic accounts of how the mind operates described at a more, abstract level and illustrated in the present discussion with the example of the MCF. The description of this theoretical framework focused on the second option treating the mind as a biological object of enquiry whereby internal states and events in the mind are viewed in a way that abstracts away from brain functioning. This functioning includes those aspects that might be regarded as pragmatic compromises that are functionally suboptimal as claimed by F&A.

An MCF-inspired approach can provide AI researchers with a picture that is clear, transparent, neuron-free and systematic showing how the mind works. If there turns out to be real potential in applying biologically-based solutions that benefit AI and especial GenAI research, this could be more useful than looking directly and solely at human brain connectivity, especially as (a) a complete picture of the highly complex wiring of the brain – the human connectome—is far from being achieved and (b) it remains questionable any way to what extent the completed connectome would provide solutions for the important questions posed here (see Nature Neuroscience, 2010). The questions for which AI researchers desire answers are more directed to how humans, that is their minds and brains, come up with creative solutions for a whole range of different problems and expend so little energy doing so. It is hard to imagine exactly how this goal can be achieved by only looking at activity at the neuro-structural level without also analysing the nature of the problems the brain is attempting to solve. A specification of the psychological aspects of problem-solving concerning the ways in which the mind approaches a task, finds solutions, uses logical and lateral thinking, deploys its memory resources and so on will, then, inevitably be part of the investigation. Problems have somehow to be identified and described if researchers have to define what they are looking for. To do this one way of other involves some kind of psychological analysis. The research into how brains solve problems will therefore always have a hybrid, interdisciplinary character involving more than Marr’s third level, whether or not an elaborated model of the mind gets used or not.

Detailed applications of the MCF approach to AI were felt to be a step beyond the intended scope of this paper and the author’s expertise. However, helpful comments from internal reviewers show some grounds for hope and, by way of exemplification, a particular line research was mentioned related to, roughly put, the problem of interpreting incoming evidence derived from the world outside and deciding what details are currently relevant given limited means of access. More specifically, this relates to modelling cognition through different modes of indistinguishability, rough set approximation, i.e., things that you can say about a given concept with more or with less certainty and how it is possible to reconcile the two perspectives, and the glueing of locally Boolean contexts into a non-Boolean lattice (Pawlak, 1982; Polkowski, 2002; Yao, 2004). Also mentioned, by this reviewer was research by Gunji and Nakamura, which follows a biologically-inspired line of thinking and tries to explain the bases of human judgement (see, for example, Gunji et al., 2016). This reviewer made an important general point namely that the proposed relationship, discussed the present paper, between the two rings, the conceptual hub and the schemas constructed from representatives in different modules, as having potential in providing an alternative to centralised cognitive architectures and restricted connectionist networks.

The question now arises as to whether the interaction between the perceptual (outer ring) and non-perceptual (inner ring) levels of processing can be seen as involving two modes of indistinguishability. In broad lines this seems reasonable as we are talking about the ways in which the mind engages online with the outside world taking first the (co) activation of representations in any one or any number of the five constructed perceptual systems. These activated representations have for example, no inherent meaning or value until a cross-modular schema is constructed online (or otherwise re-activated unmodified) that also includes associated representations on the inner ring now representing a more complex, enriched interpretation of whatever is going on outside and centred on the conceptual system.

### 5.2 The potential applicability of evolved software

It is perhaps appropriate now to close with a summary of MCF features that might plausibly function as design principles for machine cognition. There are two parts to this summary, a speculative and a more practical part. The speculative part can act as a final footnote to this discussion section. It concerns the thorny question of machine consciousness, topic that is difficult to avoid completely given the interest it continues to arouse and despite the fact that there is no principled test for detecting it in systems run by AI and especially the fact that its true nature in humans simply remains unresolved, perhaps permanently. However, the MCF does have at least some specific things to say about consciousness with ‘awareness’ being treated as synonym of consciousness. The more practical part will follow in the concluding section below.

Conscious processing in the MCF is described as involving many representations in many modules. The conditions of any kind of consciousness include the combined activation of perceptual representations on the outer ring at very highly levels of activation. The lowest perceptual level of awareness, commonly referred to, for example, as ‘phenomenal’ or ‘primary’ consciousness is shared by many species (Damasio, 1999; Edelman and Tononi, 2000): it would, in a robot equipped with environmental sensors, be difficult to reproduce the human experience of awareness. Being able to sense the environment and response to it, by itself only mimics the kind of consciousness that a simple organism might have when negotiating with its own environment. To achieve the higher, ‘thinking’ level of consciousness that humans possess, it would need to have available some equivalent to the array of perceptual representations in the mind’s five perceptual stores plus the ability of the human conceptual system to harness this resource and somehow translate abstract conceptual representations into perceptual terms so that humans experience the representations as perceptual phenomena as when we visualise in our minds an idea like a plan or a travel itinerary or do mental arithmetic. It is very hard to imagine how this could be achieved. As it is, machine intelligence produces solutions to problems, without consciousness, that human designers may or may not have consciously programmed it to provide and which in any case they can consciously interpret and utilise.

## 6 Conclusion

There are a number of different sources for AI researchers to approach the quest for biological inspiration. The large majority focus on brain machinery at Marr’s third, implementation level of analysis. There are different views about the usefulness of this strategy and the debate continues. This suggests not so much that the quest for inspiration from neural machinery be abandoned but rather supplemented by another approach which involves both detailing cognitive representations at Marr’s intermediate level as well as the level at which handles how these representations are deployed to solve problems at his first, computational level.

The present suggestion involves taking seriously models of the mind that abstract away from the neural level and look more closely at biological systems at more abstract levels. With so much interest in and focus on the physical brain, there are not so many suitable descriptions of the mind at levels above the implementational level of analysis, both treating the mind as an explicitly biological phenomenon and also explaining it in sufficient detail to qualify as a potential source of inspiration for GenAI: the MCF provides an example. Here, features that could plausibly function as design principles for machine cognition and which have been touched on at various points in the above discussion, are summarised below. In the MCF, these mental principles, some of which may reflect certain characteristics already present both in models of human cognition as well as approaches to machine cognition, have to be understood in terms of how they work together in the mind as a whole. When designing a, MCF inspired biological approach to machine cognition, even where the viability of using the MCF its account, such as it is, of conscious processing has been dismissed, an AI designer assessing the applicability of any other MCF feature would still need to keep in mind its interdependence with other MCF features.

Firstly, there is the permanent network of specialised modules, each of which have a unique function in the mind and each of which contain representations that are formed using a code that is unique to that module. The execution of virtually every task, every attempted resolution of the problems with which the mind is continually dealing with, involves more than one module and usually many modules. This provoked the question, how can incompatibly coded representations be used together in any kind a collaborative enterprise? The answer has to do with the fact that, following the MCF model, representations get activated. In other words they appear in the working memory of whatever modules they happen to belong to and in this activated (working memory) state, new associations can be formed across modules or, where already formed, an activated representation will cause the coactivation of any other associated representations in other modules. In practise this means that mental traffic is characterised by countless networks of association (schemas) that are getting activated together all the time in response to particular tasks. Code compatibility is not involved. Schemas are saved and reused and revised through the individual’s lifetime and continue to be available in varying degrees according to the regularity and frequency with which they are activated.

Secondly, a module, although it comes with its own inbuilt processing principles, does not have an empty store waiting for experience to provide the material with which to create new representations: each module has, in addition, its inherited toolkit as well with a set of ‘primitive’ representations along with some ready-made cross-modular associations including various default values via links with the affective system.

Thirdly, the mind is run as a heterarchy with no stable central executive and where priorities at any given moment are set on the basis of both current internal and external circumstances which together form the current context as the mind has interpreted them. In the competition ultimately determining which of the many co-activated candidates will end up contributing to the resolution of tasks, this combined context provides the background that leads to adjustments of the current values of representations and schemas. Within this heterarchical framework, many hierarchical procedures may be involved, for example a given, currently activated goal will have, associated and coactivated along with it, a set of subgoals.

The above three basic characteristics of MCF’s evolved software, its particular version of modularity, its inherited toolkits and its heterarchical organisation are features that may be worth considering by AI designers as productive candidates for translation into machine cognition.

## Data Availability

The original contributions presented in the study are included in the article/supplementary material, further inquiries can be directed to the corresponding author.

## Footnotes

1
https://www.cognitionframework.com
